# Rice Protein-Fortified Gummy: The Physicochemical, Bioactive and Sensory Evaluation

**DOI:** 10.3390/foods15173148

**Published:** 2026-09-04

**Authors:** Tarathep Siripan, Hua Li, Sirithon Siriamornpun

**Affiliations:** 1 Department of Food Technology and Nutrition, Faculty of Technology, Mahasarakham University, Kantarawichai, Maha Sarakham 44150, Thailand; 67010853501@msu.ac.th; 2 Department of Cuisine and Nutrition, Yangzhou University, Yangzhou 225127, China; lihua216@yzu.edu.cn; 3 Key Laboratory of Chinese Cuisine Intangible Cultural Heritage Technology Inheritance, Ministry of Culture and Tourism, Yangzhou 225127, China; 4 Research Unit of Thai Food Innovation (TFI), Mahasarakham University, Kantarawichai, Maha Sarakham 44150, Thailand

**Keywords:** Khao Dawk Mali 105, plant protein, antioxidant activity, electronic nose, electronic tongue, development, functional food

## Abstract

This study aimed to develop functional gummies enriched with protein from fully ripe (RRP) and green (GRP) Khao Dawk Mali 105 rice. Water in the standard formulation was replaced with protein extracts at 20–100% levels. The final protein content was significantly increased with increasing protein substitution, with a maximum of 12.63 g/100 g in the GRP-100 formulation. Higher protein levels generally decreased gel hardness, but a low substitution level (20%) significantly improved desirable textural properties such as springiness and chewiness. Meanwhile, moderate substitution (40%) maintained optimal structural integrity and achieved the best sensory preference within the RRP group. Bioactive evaluations showed GRP-100 possessed the highest total phenolic and flavonoid contents, alongside superior antioxidant capacity (DPPH and FRAP). Furthermore, GRP-100 achieved the highest overall consumer preference score (8.03). Moreover, electronic nose and tongue evaluations revealed that GRP formulations (especially GRP-100) exhibited the most intense and complex aroma profiles. This robust aroma may have contributed to masking bioactive-related bitterness and astringency, creating a balanced flavor that maximized consumer preference. All formulations showed good microbiological stability after 28 days of storage under refrigeration. GRP-100 provided the greatest improvement in protein enrichment, antioxidant capacity and overall consumer acceptance, whereas moderate substitution levels better preserved textural properties.

## 1. Introduction

Rice (*Oryza sativa* L.) is a staple food in Thailand and a major agricultural export that significantly contributes to the country’s economy. It is also the primary staple food for more than 50% of the global population, particularly in Asia [1,2]. Although mainly recognized as a carbohydrate source (75–80%), rice also contains protein (6–7%), fat (2–3%), dietary fiber (3–4%), vitamins, minerals, and trace phenolic compounds [1]. Rice protein has garnered growing scientific interest due to its favorable nutritional and functional properties. It possesses a well-balanced amino acid profile, high digestibility, and a protein efficiency ratio comparable to other plant-based proteins such as soy [2,3]. Analytical studies indicate that rice protein extracts contain approximately 78% total amino acids, with essential and branched-chain amino acids accounting for 36% and 18%, respectively. Particularly, rice protein is naturally free from common allergens including lactose, gluten, and soy and has shown high tolerance in individuals with cow’s milk protein allergy [4]. Its hypoallergenic and plant-based nature makes it suitable for allergen-free and vegan food formulations [5,6]. Functional properties and amino acid bioaccessibility can also be enhanced through processing methods such as thermo-extrusion [7]. Furthermore, compared to animal-derived proteins, rice protein offers a lower environmental footprint, supporting the development of sustainable, ethical food systems [8].

Protein can be extracted from rice using physical, chemical, or enzymatic methods, each with specific advantages and limitations [9]. Biological methods utilizing enzymes (amylases, proteases, carbohydrases) significantly enhance extraction efficiency by breaking down polysaccharide-protein matrices [10]. Although enzyme costs can be a consideration, enzymatic approaches offer superior preservation of protein functionality and bioactive properties. Furthermore, intact rice protein naturally exhibits poor solubility at neutral pH; however, enzymatic hydrolysis effectively cleaves large protein molecules into smaller peptide fragments and increases the exposure of ionizable and hydrophilic groups. This structural modification significantly improves the water solubility of the resulting protein hydrolysate at neutral pH, making it highly advantageous for formulations in liquid and beverage-based functional foods [11,12].

Gummy candies are very popular confectionery products. They are generally characterized by their high carbohydrate content, chewy texture and sweet taste. They are generally consumed by all demographics and especially children, adolescents and working adults. The typical gummy formulation is mainly based on sucrose, glucose syrup and animal-derived gelling agents such as gelatin [13]. However, the confectionery market has undergone a major transformation due to the changing consumer choices towards healthier eating habits. There is an increasing demand for functional gummies that offer more than just basic calories and deliver health benefits [14]. This trend has led to innovations in the food industry, such as sugar reduction, addition of vitamins and proteins [15], and substitution of ingredients with plant-based alternatives for the health-conscious consumer.

To address this trend and enhance the nutritional profile and functional value of traditional gummies, the present study proposes the incorporation of rice protein, a hypoallergenic and highly digestible plant-based alternative, into gummy formulations. Therefore, the objective of this research is to develop a novel gummy product enriched with protein extracted from Khao Dawk Mali 105 rice (KDML 105) and to comprehensively evaluate its physical, chemical, and sensory properties.

## 2. Materials and Methods

### 2.1. Materials and Chemicals

Rice samples: KDML 105 rice was harvested in Roi Et Province, Thailand, at two stages green (GR; 22–28 days post-pollination) and fully ripe (RR; 29–35 days post-pollination) and kept at 4 °C. Enzymes and basic chemicals: Food-grade protease (80,000 IU/mL) and α-amylase (3000 IU/mL) were purchased from KANEZYME (Bangkok, Thailand). Food-grade sodium hydroxide was supplied by Krungthepchemi (Bangkok, Thailand). Reagents and standards: Sigma-Aldrich Co. (St. Louis, MO, USA) provided all analytical and HPLC-grade reagents, including amino acid standards of ≥90% purity.

### 2.2. Preparation of Protein Extract

Lipid extraction for the two rice maturity stages adapted the procedure outlined by Wattanasiritham et al. [16]. The process involved sieving the ground rice through a 60-mesh screen, followed by a 1-h extraction with hexane (1:3 *w*/*v*) under agitation. A spiral evaporator under a fume hood was utilized to air-dry the mixture and eliminate residual hexane. The defatted powder was subsequently reground, passed through the sieve once more, and preserved in aluminum bags with desiccant at 4 °C for future experiments. Despite the initial defatting step, trace amounts of strongly bound structural lipids might have co-extracted with the proteins.

Rice samples of both growth stages were extracted for protein using the method of Siripan et al. [10]. The rice samples 1 g: water 10 mL were first incubated at 50 °C with 0.04% *v*/*v* of protease under continuous shaking (150 rpm, 30 min). Next, α-amylase (0.5% *v*/*v*) was introduced, followed by another 30 min of shaking. After filtration, the protein content of the extracts was measured via the Kjeldahl method (conversion factor = 5.95) [17], resulting in 18.04 g/100 mL for RR and 22.28 g/100 mL for GR. The samples were kept at 4 °C until needed. This is consistent with the report by Siripan et al. [10], which stated that the use of protease and α-amylase enzymes can help extract more proteins.

### 2.3. Preparation of Rice Protein Gummy

Protein extract solution from KDML 105 rice in two growth stages was utilized to develop protein-fortified gummy products. The base formulation was adapted from Charoen et al. [18], consisting of 33% white sugar, 33.5% glucose syrup, 8% gelatin, 1% citric acid, and 24.5% water. In the development of the gummy products, the extracted protein solution from jasmine rice was used to partially or completely replace the water content in the standard formula. The modified formulations were prepared, in which 100%, 80%, 60%, 40% and 20% of the water volume was replaced by the protein solution, as shown in Table 1. These protein-substituted formulations were then compared with the standard formulation to assess their suitability for further product development.

### 2.4. Characterization of Rice Protein Gummy

#### 2.4.1. Measurement of Color, Total Soluble Solids (TSS), pH, and Syneresis

The surface color of the produced gummies was measured according to the following procedure. The color parameters (L*, a* and b*) of the gummy samples were measured using a Chroma Meter (CR-410, Konica Minolta Sensing Inc., Osaka, Japan) after calibration with a standard white ceramic plate (Y = 87.0, x = 0.3165, y = 0.3235).

Total soluble solids (TSS) were determined using a digital refractometer (model PAL-1, Atago Co., Ltd., Tokyo, Japan). A small amount of the gummy sample was homogenized and filtered through cheesecloth to obtain a clear solution. The filtrate was placed on the prism of the refractometer, and TSS was measured at 25 °C. Results were expressed in degrees Brix (°Brix). Measurements were performed in triplicate for each sample [19].

For pH measurement, 5 g of the gummy sample was mixed with 45 mL of distilled water and homogenized for 1 min using a household food blender (Panasonic Food Processor and Blender Model MX-GM1011, Panasonic Corp., Osaka, Japan) following the method of Renaldi et al. [20], with slight modifications. The pH was measured using a pH meter (model FiveEasy F20, Mettler-Toledo GmbH, Greifensee, Switzerland).

The syneresis analysis was performed using the method of Akhavan Mahdavi et al. [21]. The gummy samples were cut into equal sizes (2 × 2 × 1.5 cm) and the initial weight was recorded. The samples were then placed in Petri dishes and kept at 4 °C for 5 days. Syneresis was calculated using the following equation:

(1) Syneresis (%) = Weight of separated liquid (g)/Initial weight of gummy (g) × 100

#### 2.4.2. Proximate Composition

The proximate composition of the samples, including moisture, protein, ash and lipid, was determined in accordance with standard methods of the AOAC [22] (methods 969.19, 920.177, 900.02 and 920.176, respectively). Total carbohydrate content was estimated by difference. All results were reported as grams per 100 g of fresh weight (g/100 g FW).

#### 2.4.3. Evaluation of Mechanical Properties

The texture of each gummy sample, cut into dimensions of 2 × 2 × 1.5 cm, was analyzed using a texture analyzer (TA-XT2i, Stable Micro Systems, Surrey, UK). Texture profile analysis (TPA) was performed using a P/35 cylindrical probe with pre-test, test, and post-test speeds of 2.0, 2.0, and 5.0 mm/s, respectively. The texture parameters measured included hardness, cohesiveness, adhesiveness, springiness, gumminess, and chewiness. Results were reported as the mean values from ten repetitions [23].

### 2.5. Structural Analysis

#### 2.5.1. Rheological Analysis

Rheological properties were measured using a controlled-stress rheometer (HAAKE MARS 40, Thermo Fisher Scientific, Karlsruhe, Germany) equipped with a 60 mm parallel plate (2 mm gap). Flow behavior was evaluated at shear rates of 0.1–100 s^−1^ at 25 ± 0.1 °C. Dynamic viscoelastic properties, including storage modulus (G′), loss modulus (G″), and loss tangent (tan δ) were determined by frequency sweep tests over 0.1–10 Hz [24].

Rheological testing for yield stress utilized a controlled-stress approach with a 60 mm parallel plate (PP60) and a 1 mm gap size. By executing a logarithmic stress sweep (0.1–200 Pa) for 120 s, the yield stress was successfully established. This value was identified by observing the shift from solid-like (elastic) to liquid-like (flow) states on a log–log graph of strain against stress.

#### 2.5.2. FTIR Spectroscopy

The functional groups of rice protein at two developmental stages were analyzed utilizing an INVENIO S/LUMOS II spectrometer (Bruker, Switzerland). Transmission spectra were collected between 4000 and 400 cm^−1^, with a resolution of 4 cm^−1^.

### 2.6. Evaluation of Antioxidant Activities

Bioactive compounds were extracted following a modified method of Siripan et al. [25]. Rice gummy (1 g) was mixed with 20 mL of 80% methanol and shaken at room temperature for 2 h at 150 rpm. The mixture was centrifuged at 4000 rpm for 10 min, and the supernatant was collected for further analysis.

The methods for analyzing bioactive compounds were adapted from Thammapat et al. [26], and the antioxidant activity from Boonarsa et al. [27].

Determination of Total Phenolic Content (TPC). Total phenolic content was determined using the Folin–Ciocalteu method [26]. Methanolic extract (0.2 mL) was mixed with 10% Folin–Ciocalteu reagent and 7% Na_2_CO_3_ solution, then incubated at room temperature for 90 min. Absorbance was measured at 725 nm, and results were expressed as mg gallic acid equivalents per 100 g dry weight (mg GAE/100 g DW).

Determination of Total Flavonoid Content (TFC). The extract was reacted sequentially with NaNO_2_, AlCl_3_·6H_2_O, and NaOH, and absorbance was measured at 510 nm. Results were expressed as mg quercetin equivalents per 100 g dry weight (mg QE/100 g DW).

Determination of DPPH Radical Scavenging Activity. The extract was mixed with 0.06 mM DPPH solution and incubated in the dark for 30 min at room temperature. Absorbance was measured at 510 nm, and antioxidant activity was expressed as mg vitamin C equivalents per 100 g dry weight (mg vitamin C/100 g DW).

Determination of Ferric Reducing Antioxidant Power (FRAP), with slight modifications. Briefly, 60 µL of extract was mixed with 180 µL of deionized water and 1.8 mL of freshly prepared FRAP reagent. The mixture was incubated at 37 °C for 4 min, and absorbance was measured at 593 nm. Results were expressed as mg FeSO_4_ per 100 g protein dry weight (mg FeSO_4_/100 g protein DW).

### 2.7. Sensory Evaluation of Gummy Samples

Sensory assessment of the gummy samples was carried out by 30 untrained panelists aged between 18 and 45 years. Prior to participation, all panelists were informed about the study objectives and provided written informed consent in accordance with the approved human research ethics protocol (No. 429-046/2025), which complies with international guidelines for sensory evaluation studies [28]. Each sample consisted of two gummy pieces (5 g per piece) and was presented in transparent plastic containers labeled with randomly generated three-digit codes. The samples were placed on white trays and allowed to equilibrate at room temperature (23 ± 2 °C) for 30 min prior to evaluation. Sensory analysis was conducted individually in a controlled sensory evaluation room. All attributes were rated using a 9-point hedonic scale, ranging from 1 (dislike extremely) to 9 (like extremely). Drinking water (low-mineral content) was provided to panelists for palate cleansing between sample evaluations.

### 2.8. Electronic Nose (E-Nose) and Electronic Tongue (E-Tongue)

#### 2.8.1. Electronic Nose Analysis (E-Nose Analysis) and Signal Acquisition

Volatile compounds of the gummy samples were analyzed using an electronic nose system (PEN3, Airsense Analytics Inc., Schwerin, Germany), following the method of Liu et al. [29] with slight modifications. The instrument is equipped with an array of ten metal oxide semiconductor sensors, as detailed in Table 2. Prior to analysis, the gummy sample was transferred into a 10 mL hermetically sealed glass vial and incubated at 60 °C for 20 min to completely melt the gummy matrix and allow volatile compounds to reach equilibrium inside the vial before measurement. The analysis was conducted under the following conditions: the carrier gas flow rate and injection flow rate were both maintained at 150 mL/min, and the sensor array was cleaned until the response signal returned to baseline. The total analysis time was 90 s, with data recorded every 0.5 s and a delay time of 210 s before data acquisition [30].

#### 2.8.2. Electronic Tongue Analysis (E-Tongue Analysis)

The taste profiles of the samples were evaluated using an electronic tongue system (SA402B, Intelligent Sensor Technology Co., Ltd., Atsugi, Japan), based on a modified method described previously [29,31]. The system measures eight taste-related parameters, including the four basic tastes (saltiness, umami, bitterness, and sourness), two mouthfeel attributes (intensity and astringency), and two aftertaste characteristics (astringent aftertaste, aftertaste-A; and bitter aftertaste, aftertaste-B). For sample preparation, 5 g of gummy was mixed with 50 mL of distilled water and homogenized thoroughly. The mixture was then centrifuged at 10,000 rpm for 5 min at 4 °C. After centrifugation, the supernatant was collected, and 40 mL of the clear solution was used for taste analysis. Each sample was analyzed four times. To reduce the effect of sensor instability, the first measurement was discarded, and the remaining three measurements were used for statistical analysis.

### 2.9. Study on the Shelf Life of Gummy Samples

The quality of gummy samples packaged in sterile plastic bags was evaluated throughout the storage period. To prevent cross-contamination, samples were individually packed for each designated sampling day, squeeze out all the air and kept in an incubator at ≤4 ± 2 °C [32], at predetermined intervals (0, 7, 14, 21, and 28 days). At predetermined intervals (0, 7, 14, 21, and 28 days), microbiological quality was assessed by determining total plate count (TPC) as well as yeast and mold populations. Microbial results were reported as colony-forming units per gram of sample (CFU/g). Due to its high moisture content, refrigeration is necessary to preserve texture and bioactive compounds, preventing spoilage.

### 2.10. Data Analysis

Data processing and statistical evaluations were performed using OriginPro 2022 (OriginLab Corporation, MA, USA) and SPSS version 23.0 (SPSS Inc., IL, USA), respectively. All data are expressed as the mean ± standard deviation (SD) of triplicate measurements. Statistical differences among treatment groups were analyzed using one-way analysis of variance (ANOVA) followed by Duncan’s multiple range test at a 95% confidence level (*p* < 0.05).

## 3. Results and Discussions

### 3.1. Evaluation of Physical Properties of Gummy Supplemented with Rice Protein Extract

The physical properties of gummies supplemented with rice protein extracts showed significant variation depending on the source of protein (RRP vs. GRP) and the percentage of protein extract substitution. Color measurements (L*, a*, b*) revealed distinct trends between the two protein sources. The incorporation of RRP generally resulted in lower L values compared to the control sample, indicating a darker gummy matrix with increasing protein substitution. Conversely, four formulations of GRP (GRP-20, GRP-40, GRP-80, and GRP-100) exhibited higher L values than the control, suggesting that GRP incorporation contributed to a lighter gummy appearance [33].

As shown in Table 3, the redness (a*) and yellowness (b*) parameters were significantly altered. The GRP groups exhibited positive a* values, particularly at 60–100% substitution, compared to the control and RRP groups, suggesting a shift toward more reddish hues. Interestingly, the RRP-100 group had the lowest L* and b* values, reflecting a darker and less yellow hue. This could be attributed to the fully ripe rice protein, which led to intensified non-enzymatic browning.

Beyond their influence on appearance, rice protein supplementation altered the physicochemical characteristics of the gummy matrix, including soluble solid content, acidity, and syneresis, reflecting changes in the interactions between rice proteins and the gelatin-based network. Total soluble solids (TSS) climbed steadily with higher supplementation, peaking in the GRP-100 formulation (41.37°Brix). This increase represents more than just a simple additive effect of dry matter; it indicates a denser, more complex solute matrix as the proteins and their associated micro-constituents integrate into the continuous phase [34]. The pH values of the gummy formulations exhibited concentration-dependent variations rather than opposing trends between the two protein sources. At lower to medium substitution levels (20–60%), formulations from both protein extracts generally became more acidic compared to the control (dropping to 3.26 in RRP-60 and 3.15 in GRP-60), which may point directly to a higher release of free amino acids and native organic acids from the grains [35]. However, at higher substitution levels (80% and 100%), both RRP and GRP samples demonstrated an upward trend in pH, rather than maintaining a highly acidic environment, reaching 3.77 in RRP-100 and 3.60 in GRP-100.

The structural implications of these chemical shifts are most obvious when considering syneresis, which varied greatly between the treatments. The lowest syneresis was obtained in RRP-20 (1.78%) which is indicative of improved water-holding capacity at lower substitution levels. However, syneresis was much higher in GRP-100 (3.12%), indicating that excessive substitution of protein decreased water retention, suggesting that high protein concentrations may interfere with hydrocolloid network formation. This behavior suggests a structural tipping point. At excessive concentrations, competitive protein-polysaccharide interactions likely disrupt the continuous hydrocolloid network, preventing optimal cross-linking and forcing water out of the interstitial spaces [36,37]. It is noteworthy that GRP-60 had relatively low syneresis (2.58%) while maintaining favorable TSS and pH, suggesting that this ratio may represent an optimal balance of stability and nutritional enhancement.

The results indicate that both the botanical origin (RRP vs. GRP) and substitution level of rice protein significantly influence the physicochemical properties of the gummy system. The GRP extracts were more effective in improving TSS and maintaining pH stability, while RRP provided superior syneresis reduction at lower concentrations. These outcomes align with previous studies emphasizing the role of the protein source and molecular composition on gel behavior [38,39].

### 3.2. Proximate Composition of Gummy Fortified with Rice Protein Extract

The proximate analysis (Table 4) clearly illustrates that incorporating rice protein extracts successfully transformed the nutritional baseline of the gummy matrix. As expected, the level of protein enrichment was directly proportional to the substitution level, increasing the protein content from a modest 7.17 g/100 g in the control to 11.59 and 12.63 g/100 g in the RRP-100 and GRP-100 formulations, respectively. It is interesting to note that at matching substitution ratios, the green rice protein (GRP) always gave a slightly higher protein payload than mature rice protein (RRP). Given that a food matrix acts as a closed mass-balance system, high protein fortification inevitably led to the natural displacement of the main bulking agents in the formulation, which led to a proportional and inevitable decrease in the total carbohydrates [40]. Beyond this primary macronutrient shift, the incorporation of the extracts introduced secondary compositional changes that offer insights into both the ingredient’s purity and its physical behavior. The steady climb in lipid and ash fractions—particularly prominent within the GRP series points directly to the residual native fats and minerals carried over during the extraction of the rice proteins [41]. In contrast, the moisture content showed a slight decrease at higher substitution levels. This reduction may be related to the enhanced water-binding capacity of the added proteins, which effectively reduces the free water content within the structural matrix of the gummies [42].

Overall, these findings indicate that the incorporation of rice protein extracts significantly enhanced the nutritional profile of the gummies most notably through protein enrichment while simultaneously modulating other fundamental compositional parameters.

### 3.3. Evaluation of Mechanical and Textural Properties of Gummy Supplemented with Rice Protein Extract

Texture Profile Analysis (TPA) is a widely applied method in the food industry for evaluating the textural properties of food products, providing critical insights into their sensory attributes [43]. The data in Table 5 comprehensively illustrates the effects of incorporating fully ripe rice protein (RRP) and green rice protein (GRP) extracts at various substitution levels (20–100%) on the textural properties of the gummies. The evaluated parameters hardness, cohesiveness, adhesiveness, springiness, gumminess, and chewiness are critical quality indicators for consumer acceptance in gel-based functional foods.

A significant reduction in hardness was observed in most protein-supplemented samples compared to the control (39.42 N) (*p* < 0.05), with the exception of the GRP-20 formulation. This softening effect became more pronounced as the substitution level increased, reaching the lowest values in the RRP-100 (15.71 N) and GRP-100 (22.82 N) formulations. This decline can be attributed to the interference of rice proteins with the gel matrix formation. The addition of protein likely hindered gel-network reinforcement and diluted the primary gelling agents, a phenomenon consistent with findings by Sabater et al. [44], who reported that plant protein fortification can weaken gel structures by disrupting polymer-polymer interactions. Unlike gelatin, rice proteins do not readily form an elastic three-dimensional network under these processing conditions.

Cohesiveness significantly increased in specific high-protein treatments, peaking in the RRP-100 sample (0.97) compared to the control (0.88) (*p* < 0.05). This suggests that protein-protein interactions at certain concentrations may enhance internal bonding within the gel matrix [45]. For adhesiveness, the control sample exhibited the most negative value (−0.34), indicating a highly sticky nature. The incorporation of both RRP and GRP significantly reduced stickiness across all treatments (ranging from −0.09 to −0.02), with GRP-100 demonstrating the least stickiness (−0.02). This highlights the capability of rice protein extracts to favorably modify the surface stickiness of the gummy matrix [46].

Interestingly, the addition of proteins, particularly at specific levels, enhanced the springiness of the gummies. Formulations such as RRP-20 (1.17) and GRP-60 (1.21) showed significantly higher springiness values than the control (0.83). This suggests that the incorporated rice proteins contribute to elastic recovery under deformation, likely due to their water-binding capacity and partial network formation capability. Similar improvements in elasticity have been documented in legume protein-fortified gels [47]. However, springiness notably dropped at the 80% substitution level for both protein types (0.81 for RRP-80 and 0.84 for GRP-80) before rising again at 100%.

Both gumminess and chewiness exhibited varied trends, with lower substitution levels generally providing higher resistance. The RRP-20 formulation demonstrated the highest gumminess (41.89 N) and chewiness (47.18 N). This implies that a low substitution level (20%) achieves an optimal balance between protein network reinforcement and gel matrix integrity. Conversely, higher protein levels (80–100%) significantly reduced both gumminess and chewiness, likely due to excessive dilution of the gelling agents and the limited cross-linking capacity of rice proteins at high concentrations [48].

In summary, when comparing the samples at equivalent substitution levels, the GRP (green rice protein) treatments generally maintained slightly higher hardness than the RRP (fully ripe rice protein) formulations at high concentrations (GRP-100 vs. RRP-100). This variation may stem from compositional differences between the extracts at different maturity stages, which influence water retention and elasticity [49]. Overall, low substitution levels (around 20%) appear most favorable for maximizing gumminess, chewiness, and springiness without overly compromising the structural integrity of the gummies. Meanwhile, higher substitution levels result in a softer, less sticky, and less chewy matrix. These findings highlight the potential of using varying levels of rice protein extracts to tailor the texture of functional gummies for specific consumer demographics, such as children or the elderly, who may prefer softer products.

### 3.4. Rheology

**Figure 1:**
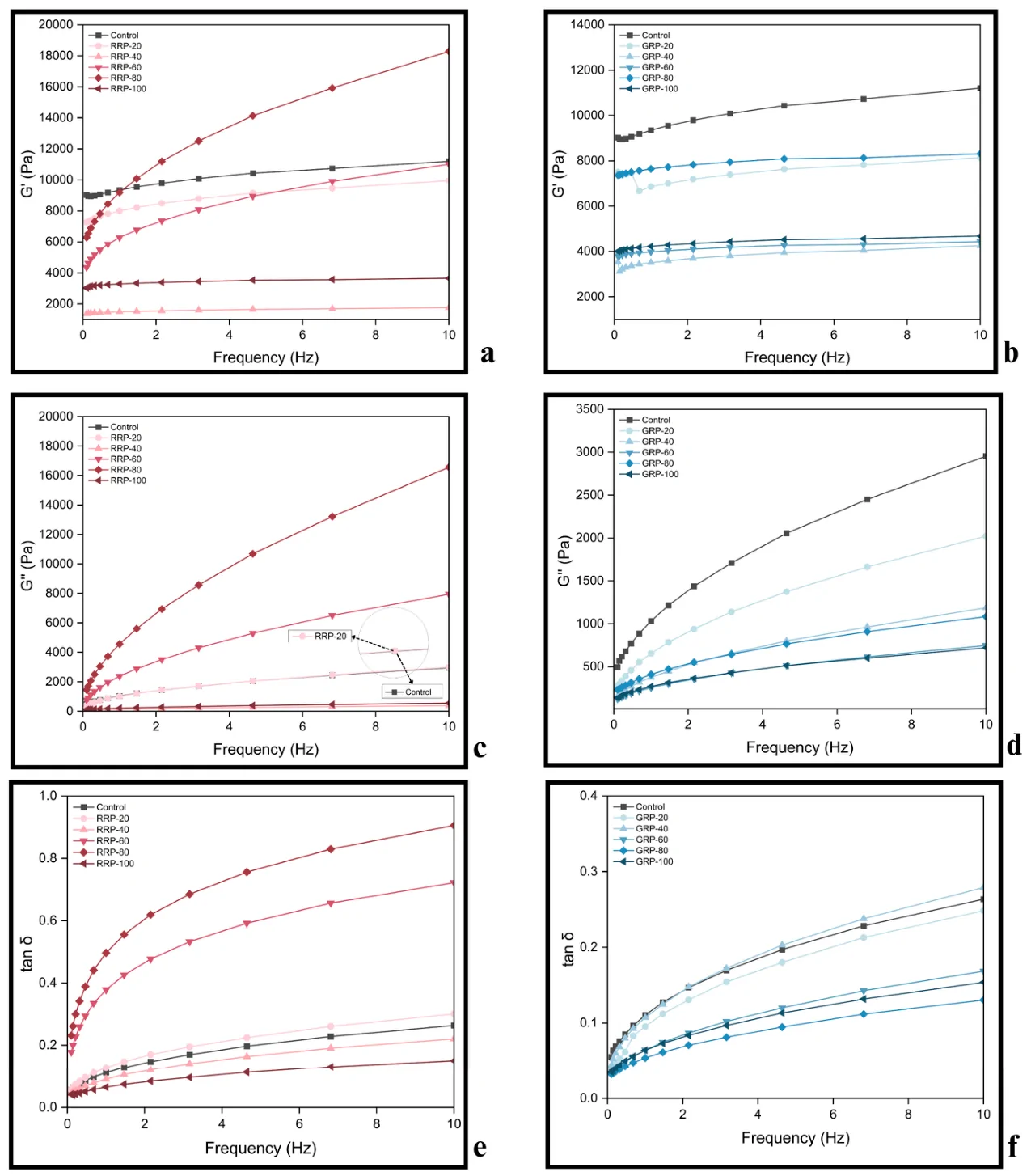
Rheological properties of rice protein gummy samples prepared with different rice protein extraction methods. RRP (fully ripe KDML 105 rice protein extract) and GRP (green KDML 105 rice protein extract). The numbers following the sample names (20–100 respectively) represent the percentage of protein extract replacing water in the formulation (%). (**a**) Storage modulus (G′) of RRP samples; (**b**) Storage modulus (G′) of GRP samples; (**c**) Loss modulus (G″) of RRP samples (the control line is hidden under the RRP20 line due to nearly identical values.); (**d**) Loss modulus (G″) of GRP samples; (**e**) Loss tangent (tan δ) of GRP samples; and (**f**) Loss tangent (tan δ) of RRP samples.

The dynamic rheological properties of the gummy products were evaluated via frequency sweep tests to assess structural stability and the time-dependent behavior of the gel networks [24]. The storage modulus (G′), indicative of the matrix is elastic character, demonstrated that the type and concentration of rice protein extracts significantly influenced structural integrity. The control formulation exhibited a highly stable, strong elastic network characterized by minimal frequency dependence and G′ values ranging from 9000 to 11,000 Pa. Conversely, the incorporation of green rice protein (GRP) generally attenuated the gel structure, as evidenced by a ubiquitous reduction in G′ (Figure 1b). While the GRP-20 formulation maintained structural integrity comparable to the control, higher GRP concentrations induced a substantial decline in G′ to approximately 4000 Pa. In contrast, the addition of fully ripe rice protein (RRP) induced a pronounced non-linear, frequency-dependent viscoelastic behavior (Figure 1a). Notably, the G′ of the RRP-60 and RRP-80 formulations increased markedly with frequency. The RRP-80 variant reached a peak G′ exceeding 18,000 Pa, suggesting a network governed by physical entanglements that lack sufficient relaxation time at high frequencies. Evaluation of the viscous response via the loss modulus (G″) further underscored these divergent microstructural impacts. All GRP-containing formulations (Figure 1d) exhibited lower G″ values than the control, reflecting a rigid network with diminished flow capacity. Conversely, specific concentrations of RRP (Figure 1c) induced a substantial elevation in viscous flow behavior. The G″ of RRP-80 reached over 16,000 Pa at 10 Hz, suggesting profound microstructural relaxation under rapid mechanical oscillation. To comprehensively assess the viscoelastic character, the loss tangent (tan δ = G″/G′) was analyzed [50]. Although all formulations exhibited tan δ < 1, confirming predominantly solid-like behavior, the GRP group maintained consistently low values (tan δ < 0.3) (Figure 1f), indicative of a robust, highly elastic network comparable to the control. However, the RRP-60 and RRP-80 formulations (Figure 1e) displayed a continuous increase in tan (δ), approaching 0.7 and 0.9, respectively, signifying a transition toward more viscous flow behavior. The strength and network characteristics of the gel (minimal vs. non-linear frequency dependence) were evaluated using a frequency sweep test via the small amplitude oscillatory rheology technique, based on the gel formation theory from the research of Osorio et al. (2007) [51], which states that gel structures exhibit higher G′ values than G″. These rheological insights are pivotal for targeted texture engineering, and dynamic rheological measurements evaluate small-amplitude deformation, whereas TPA measures large deformation. The incorporation of GRP effectively preserves the characteristic elastic properties of traditional gummies. Meanwhile, RRP supplementation, particularly at 60–80% replacement levels, generates a highly frequency-dependent matrix. This structural modification may manifest as increased stickiness or deformability during mastication, thereby offering a novel approach for modulating the mouthfeel of gummy products.

### 3.5. Structural Characterization by FT-IR Spectroscopy

**Figure 2:**
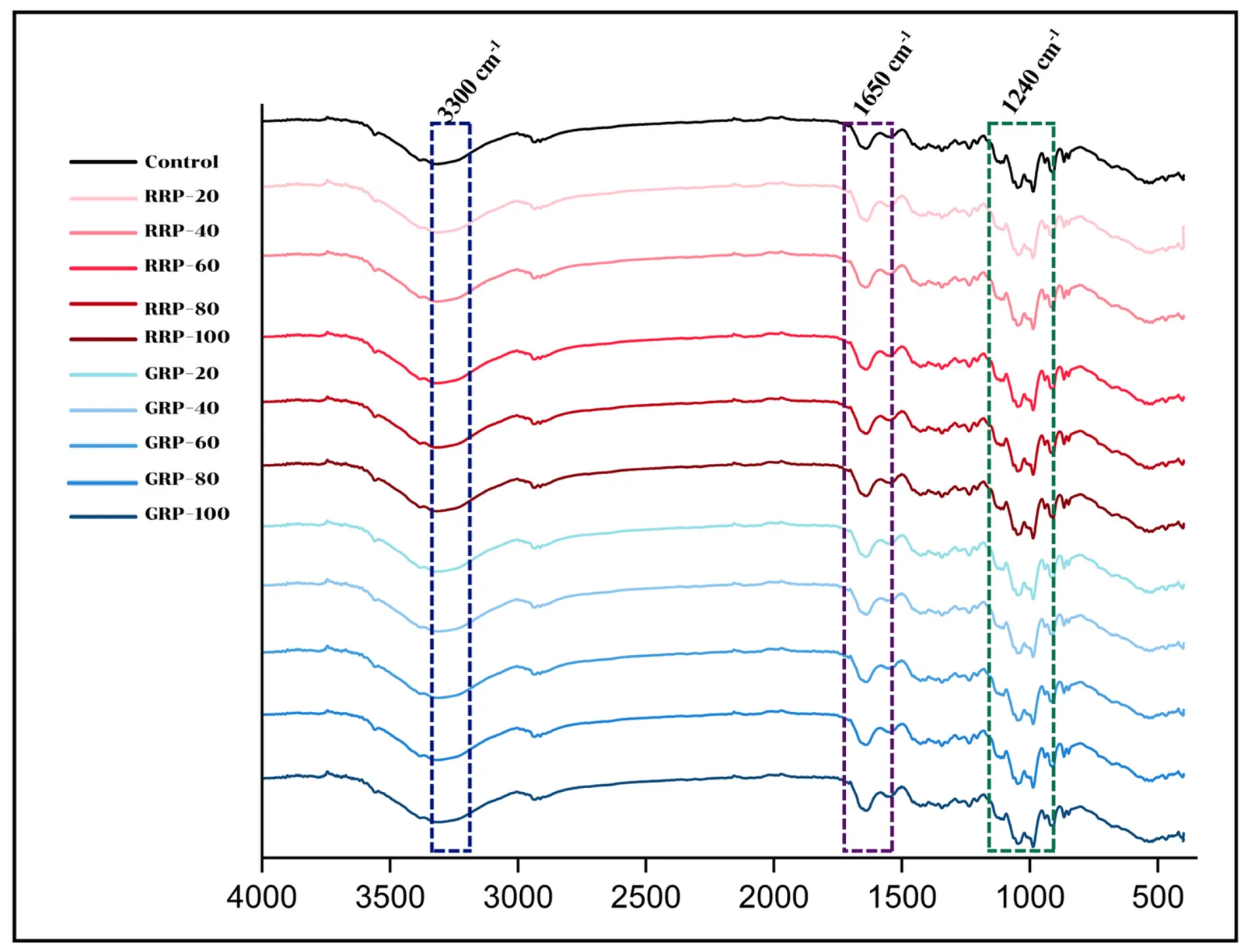
FTIR spectroscopy was utilized to characterize rice proteins derived from various extraction methods, as well as to evaluate the spectral features of the formulated rice protein gummy. RRP (Fully ripe KDML 105 rice protein extract), GRP (Green KDML 105 rice protein extract), numbers after sample names (20–100 respectively) are the amount of protein extract water replacement in different ratios to water ratio in the formula (%).

The structural characteristics of rice protein extracted using different methods were analyzed using Fourier Transform Infrared (FTIR) spectroscopy. The FTIR spectra of rice protein gummy samples are shown in Figure 2. In general, all the samples showed a similar pattern of absorption. This suggested that the functional groups of proteins were maintained at major levels regardless of the extraction method or substitution level. Broad band in the region 3200–3500 cm^−1^ is due to O-H and N-H stretching which reflect hydrogen bonding in proteins and residual moisture [52]. The peaks in the 2800–3000 cm^−1^ region are due to C–H stretching vibrations.

The characteristic amide bands were observed clearly. The Amide I band (ca. 1600–1700 cm^−1^) is dominated by C=O stretching and is often used for the assessment of protein secondary structure. The Amide II band (ca. 1500–1600 cm^−1^) is associated with N-H bending and C-N stretching [52,53]. These bands were observed in all the samples with a slight change in intensity, indicating a minor structural difference depending on the extraction. These bands appeared in all samples with slight variations in intensity, suggesting minor structural differences depending on the extraction method and protein content. In the fingerprint region (approximately 800–1200 cm^−1^), several peaks were detected, which may be attributed to C–O, C–C, and C–O–C stretching vibrations, possibly related to polysaccharides or interactions between protein and other components in the gummy matrix [54].

Overall, the FTIR results indicate that the protein structure remained relatively stable across different treatments, although slight shifts in peak intensity suggest that both the extraction method and the level of protein substitution may influence molecular interactions within the product.

### 3.6. Biological Properties of Gummy

The enrichment of gummies with rice protein extract significantly enhanced their biological activities, as demonstrated by increased total phenolic content (TPC), total flavonoid content (TFC), DPPH radical scavenging activity, and ferric reducing antioxidant power (FRAP) (Table 6). These enhancements were influenced by both the botanical origin (RRP vs. GRP) and the concentration of the protein extract used in the formulations.

Regarding TPC, the supplemented samples exhibited significantly higher values than the control (134.83 mg GAE/100 g DW), with the GRP-100 group showing the highest value (1099.45 mg GAE/100 g DW). The substantial increase in TPC in the GRP samples is consistent with previous reports indicating that green rice contains higher levels of bound and free phenolic acids, particularly ferulic acid and *p*-coumaric acid, which contribute to its antioxidant potential [55,56]. The progressive increase in TPC with increasing protein extract levels supports the hypothesis that water-substituted protein extracts serve as rich carriers of phenolic compounds extracted from green rice during enzymatic hydrolysis [57].

TFC followed a similar trend to TPC, with the highest content found in GRP-100 (486.08 mg QE/100 g DW), significantly higher than that of RRP-100 (340.32 mg QE/100 g DW). These results suggest that flavonoid concentration is strongly influenced by the rice maturity stage, with immature green grain (GRP) extracts retaining more bioactive flavonoids such as tricin and apigenin derivatives [58]. The enzymatic extraction method may have further enhanced flavonoid recovery by breaking down cell wall structures, facilitating the release of bound phenolics, as demonstrated in legume-based antioxidant studies [59].

The antioxidant activity, as measured by the DPPH radical scavenging assay, showed a notable increase in the enriched samples, particularly at higher substitution levels. GRP-100 recorded the highest DPPH activity (47.70 mg Vitamin C/100 g DW), which was approximately ten times greater than that of the control. These results reinforce the findings by Martillanes et al. [60]. Furthermore, the RRP samples also showed increased DPPH activity with rising protein extract concentrations up to 80% (44.51 mg Vitamin C/100 g DW) before a slight decline at 100% substitution. Overall, their maximum values remained lower than those of their GRP counterparts, possibly due to lower overall flavonoid diversity.

FRAP results aligned with those of DPPH and TPC, showing enhanced reducing power in all enriched samples. GRP-100 had the highest FRAP value (192.24 mg FeSO_4_/100 g DW), suggesting a strong electron-donating capacity that correlates with its phenolic and flavonoid contents. This result is in line with studies by Ti et al. [61] and Zhou et al. [56], who identified rice and its germ as potent sources of antioxidants capable of contributing to redox stability in functional foods.

Collectively, the results suggest that GRP extracts, particularly at 100% water replacement, are superior to RRP in enhancing antioxidant capacity due to their richer composition of phenolic and flavonoid compounds. However, RRP samples also provide significant bioactivity enhancements, indicating their potential as antioxidant fortifiers. These findings are consistent with the broader literature on the health-promoting properties of rice-derived ingredients, confirming their viability in functional food product development [62,63].

### 3.7. Sensory Evaluation 

#### 3.7.1. Consumer Taste Testing

**Figure 3:**
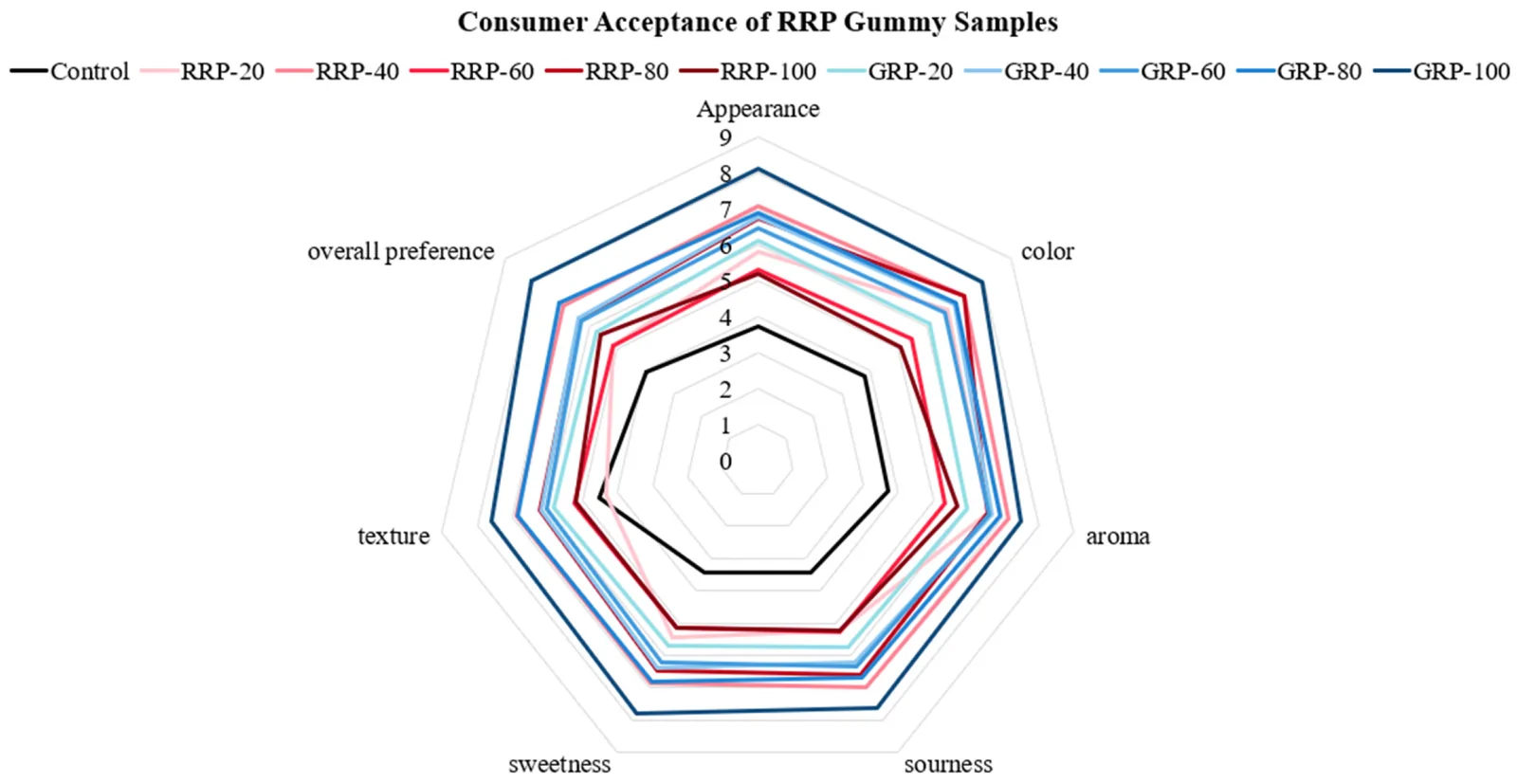
Sensory evaluation of gummy jellies fortified with rice protein extract. The sensory attributes (appearance, color, aroma, sourness, sweetness, texture, and overall preference) are shown for formulations containing fully ripe KDML 105 rice protein (RRP) and formulations containing green KDML 105 rice protein (GRP). The numbers following the sample abbreviations (20–100 respectively) denote the ratio of water replacement by the protein extract (%). The control represents the standard gummy without rice protein addition.

The sensory characteristics of gummy samples prepared with different formulations were evaluated by an untrained consumer panel (*n* = 30, aged 18–45 years) using a 9-point hedonic scale [64,65]. The attributes assessed included appearance, color, aroma, sourness, sweetness, texture, and overall preference. The results are presented in Figure 3.

Regarding the RRP samples, all sensory scores were significantly higher than those of the control group. The control sample received the lowest scores across all attributes, with an overall liking score of 3.96, indicating low consumer acceptance. The sensory quality, particularly in terms of color, aroma, and overall liking, was notably improved by the addition of RRP. Among the RRP formulations, RRP-40 achieved the highest overall preference score (6.93), alongside favorable ratings for appearance (7.06), color (7.36), aroma (7.13), and sweetness (6.86). Increasing the RRP level beyond 40% did not further improve acceptability. In fact, the RRP-60 and RRP-100 samples exhibited slightly reduced scores, especially for appearance and flavor attributes. This suggests that excessive addition may disrupt the sensory balance, likely due to the characteristic off-flavors or grittiness commonly associated with high concentrations of plant proteins [66].

A similar trend was observed for the GRP samples, but they generally scored higher than the RRP group. All sensory attributes of the GRP formulations were significantly higher than the control. GRP-100 had the highest scores, suggesting the highest overall liking (8.03) and better scores for appearance (8.10), color (7.96), aroma (7.50) and sweetness (7.80). The increasing GRP levels still seemed to be at an acceptable concentration relating to a better sensory acceptance, which means that GRP improved the product quality without negative effects at higher concentrations. The higher acceptance might be explained by the specific aromatic profile of young green rice which could better mask off-flavors than fully ripe rice proteins [2,10,25].

Texture scores across all samples showed moderate variation, ranging from 4.33 to 7.60. Both RRP and GRP formulations generally improved the texture relative to the control, although no clear linear trend was observed. This variation may stem from differences in the gel structure or the water-binding properties of the added ingredients. Previous studies have demonstrated that the inclusion of protein extracts can modify the hydrocolloid network, altering the water-binding capacity and subsequently affecting the chewiness and firmness of gummy gels [67]. Overall, the results indicate that incorporating rice protein ingredients significantly improved the sensory properties of the gummy products. RRP-40 emerged as the most preferred formulation within the RRP group, whereas GRP-100 was the most highly accepted sample across all treatments. These findings demonstrate that GRP possesses a higher potential for enhancing both the sensory quality and consumer preference of functional gummy applications.

#### 3.7.2. Prediction Based on Random Forest (Electronic Nose (E-Nose) and Electronic Tongue (E-Tongue))

##### Heatmap Chart of the Aroma Profiles Across Different Treatments Based on Electronic Nose (E-Nose) Analysis

**Figure 4:**
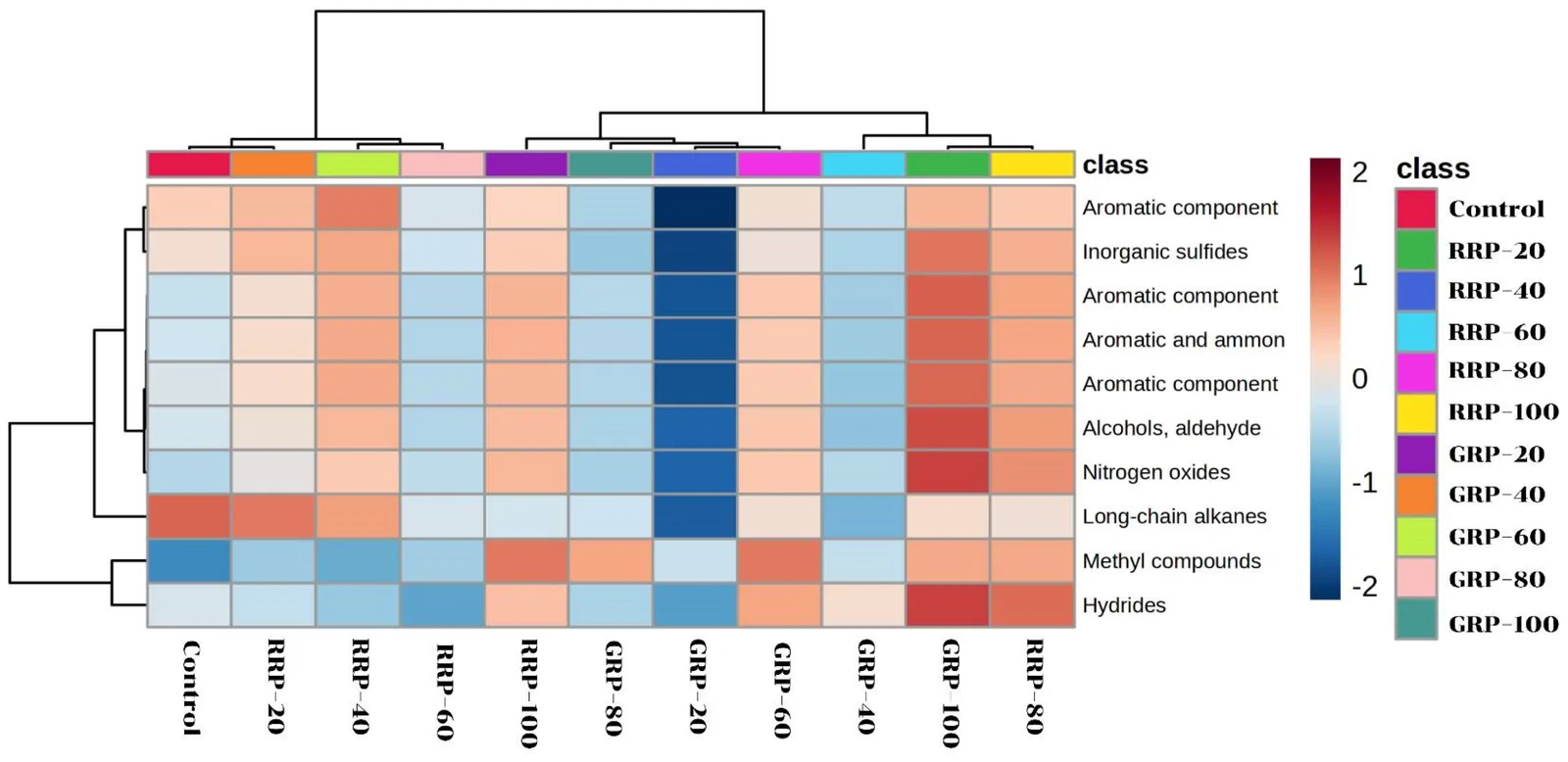
Heatmap chart showing the odor patterns of samples analyzed using the electronic nose (E-nose). The numbers on the axis represent the sensor response values to 10 groups of volatile compounds in each experimental set. RRP (fully ripe KDML 105 rice protein extract) and GRP (green KDML 105 rice protein extract). The numbers following the sample names (20–100 respectively) represent the percentage of protein ex-tract replacing water in the formulation (%).

Electronic nose technology has been widely used as a rapid and non-destructive tool for evaluating food aroma because the sensor array can generate characteristic odor fingerprints that reflect differences in volatile compound composition [29]. Figure 4 presents the aroma profiles of the control and treated samples determined by the e-nose (PEN3 system) using ten metal oxide semiconductor (MOS) sensors.

While the control sample presented a relatively muted aroma profile with the lowest overall sensor responses, the protein-fortified formulations painted a much more vibrant picture. The GRP-100 treatment, followed closely by GRP-80 and RRP-100, exhibited the most robust and complex volatile signatures. This concentration-dependent surge in sensor signals strongly suggests that the rice protein extracts act as active precursors for aroma development, rather than just inert nutritional fillers.

A closer examination of the sensor behavior reveals elevated responses from W1C, W5S, W3C, W2S, and W2W across the treated samples. Because these specific sensors are highly sensitive to broad classes of aroma-active compounds—including aromatics, nitrogen oxides, alcohols, aldehydes, ketones, and sulfur-containing volatiles [68], their heightened activity points to a highly diverse pool of volatiles. The generation of these compounds is likely linked to the inherent chemical composition of the rice extracts, potentially involving mild lipid oxidation or Maillard-driven interactions between the added proteins and the gummy matrix during processing.

Ultimately, these instrumental readings bridge the gap between chemical composition and human perception. The fact that the GRP-100 formulation exhibited the most intense odor fingerprint aligns perfectly with our sensory evaluation data. It becomes evident that higher supplementation levels do not simply add “more” scent; rather, they introduce a more complex aromatic profile that significantly enhances the specific olfactory attributes and flavor perception of the final product [69].

##### Heatmap Chart of Taste Profiles Across Different Treatments Based on Electronic Tongue (E-Tongue) Analysis

**Figure 5:**
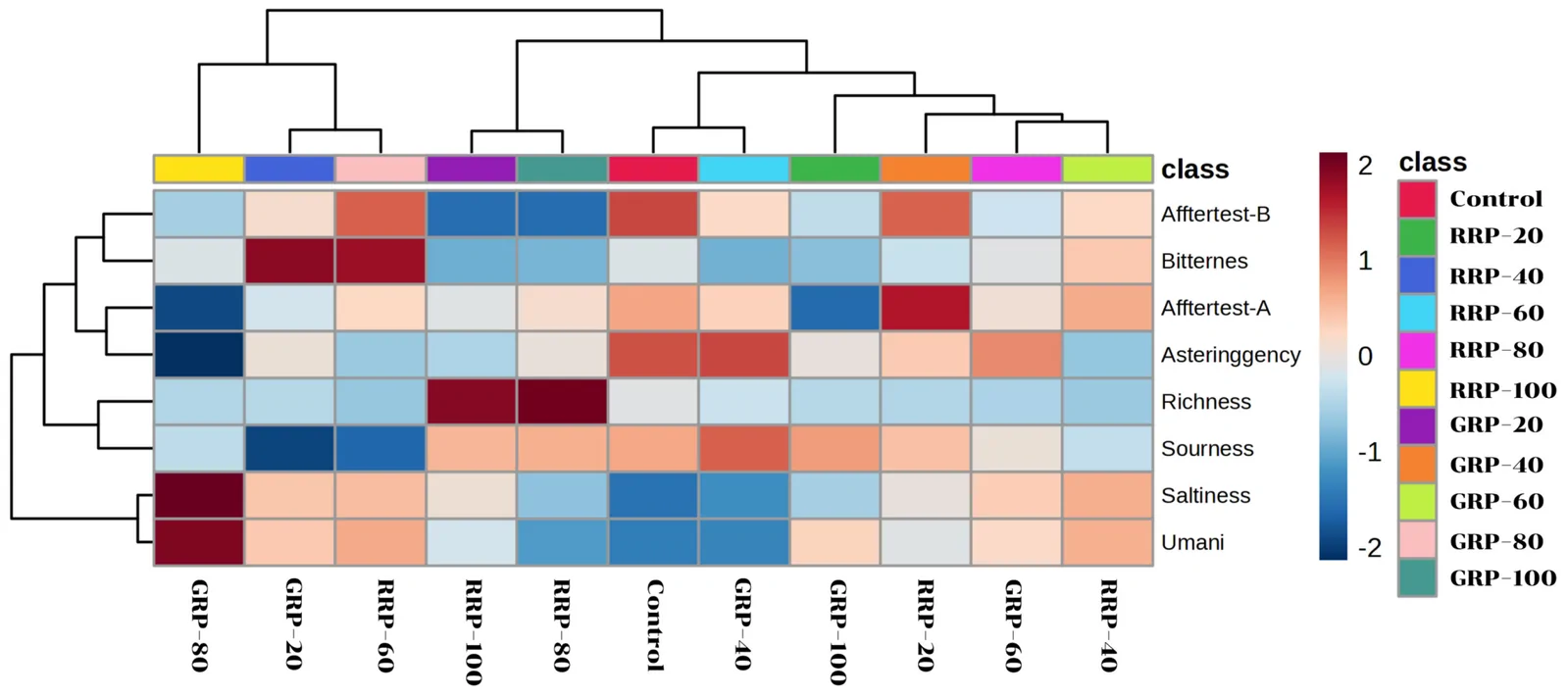
Heatmap chart illustrating the taste profiles of samples analyzed using electronic tongue (E-tongue). Numbers on the axis represent the level of response to eight taste characteristics: sourness, bitterness, astringency, aftertaste (A, B), umami, richness, and saltiness in both the control and experimental groups. RRP (fully ripe KDML 105 rice protein extract) and GRP (green KDML 105 rice protein extract). The numbers following the sample names (20–100 respectively) represent the percentage of protein ex-tract replacing water in the formulation (%).

Electronic tongue systems employ lipid membrane sensors that mimic human taste perception and have been widely applied for objective characterization of food taste attributes [70]. The heatmap (Figure 5) shows that rice protein addition, particularly green rice protein (GRP) extract, altered the taste architecture of the gummies. The added proteins were not just a passive macronutrient, but resulted in measurable changes in the chemical makeup and sensory profile of the product.

The major characteristic of all the formulations was bitterness and the intensity increased proportionately with the level of supplementation. GRP-80 and GRP-100 showed the strongest bitterness, with increases in astringency and a lingering bitter aftertaste (aftertaste-B). Biochemically this is a classic response to the ingestion of plant derived bioactives. The phenolic compounds and low molecular weight peptides native to the green rice extract are known to contribute to these distinct flavor notes [71,72]. The heightened astringency, in particular, points directly to the interaction and subsequent precipitation of salivary proteins by these polyphenols, creating the characteristic drying sensation [73]. Moreover, the elevated aftertaste responses suggest that the incorporation of rice-derived ingredients influenced taste persistence, an important factor affecting overall consumer perception. In contrast, all samples exhibited negative response values for sourness, aftertaste-A, umami, richness, and saltiness, although the magnitude of these responses varied among treatments. The control and lower supplementation levels generally showed lower umami and richness responses, whereas GRP-80 and GRP-100 exhibited slightly enhanced richness and umami characteristics. These changes may reflect the presence of amino acids, peptides, and other flavor-active compounds inherently found in green rice [74], which are known to contribute to savory taste perception and mouthfeel.

When looking at the broader picture, GRP-100 undeniably delivers the most complex sensory signature. What is remarkable here is that despite the sharp increases in bitterness and astringency, the overall taste matrix does not become overwhelmed or disjointed. Instead, the green rice ingredients function as dynamic flavor modulators, contributing taste-active compounds that deepen the structural complexity and maintain a robust, balanced flavor profile in the final gummy product.

One of the most intriguing findings from this study is the apparent paradox between the instrumental flavor profile of the GRP-100 formulation and its consumer acceptance. While the E-tongue detected elevated bitterness and astringency—attributes typically expected to negatively impact preference, the sensory panel actually awarded GRP-100 the highest overall liking score.

This behavior highlights a fascinating dynamic often observed in complex food matrices, particularly in protein-enriched functional foods. The E-nose data revealed that GRP-100 possesses a remarkably complex volatile profile. When this enhanced aroma complexity interacts with the slight increases in richness and umami responses picked up by the E-tongue, it appears to effectively mask or compensate for the bitter and astringent notes. Instead of rejecting the product based on a single negative taste attribute, consumers responded to the harmonious integration of aroma, taste, and mouthfeel, resulting in a well-rounded and appealing sensory experience.

From a compositional standpoint, these flavor shifts are undoubtedly driven by the unique matrix of the green rice protein extract. The variations in specific peptides, phenolic compounds, and free amino acids inherent to rice at this maturity stage act as key flavor-active constituents. Ultimately, the strong agreement between our instrumental tools and human panelists not only validates the predictive power of the E-nose and E-tongue but also confirms a practical application: green rice protein can successfully elevate both the nutritional profile and flavor complexity of gummy products without compromising consumer appeal.

### 3.8. Shelf Life of Gummy

Assessing the total microbial count, including yeasts and molds, is a critical indicator in shelf-life testing to prevent spoilage, maintain product quality, and ensure consumer safety throughout the storage period [75]. Table 7 displays the total plate count of the gummy samples during refrigerated storage (≤4 °C) over 28 days. No microbial growth was detected (n.d.) in any of the formulations during the first 14 days, indicating excellent microbiological stability in the initial phase. By day 21, microbial counts emerged at very low levels (0.33–1.00 CFU/g) across all samples, with no significant differences observed among the treatments (*p* > 0.05).

After 28 days, the total plate count values slightly increased in all formulations, ranging from 1.00 to 2.66 CFU/g. The highest microbial load was observed in GRP-100 (2.66 CFU/g), while the lowest was recorded in RRP-20 and GRP-20 (both 1.00 CFU/g). Although significant differences (*p* < 0.05) were noted among certain formulations at day 28, the overall microbial loads for all samples remained exceptionally low and well within safe consumption limits. Based on the Thai Food and Drug Administration (Thai FDA) Notification No. 416 (B.E. 2563) [76], and Codex Alimentarius guidelines for confectionery products [77], the acceptable regulatory limits for total plate count and yeast/mold are generally set at < 10,000 CFU/g and < 100 CFU/g, respectively. The maximum counts observed in our formulations at day 28 (2.66 CFU/g for TPC and 4.67 CFU/g for yeast and mold) were well below these critical safety thresholds, confirming the excellent microbiological quality of the products. This high stability can be primarily attributed to the refrigerated storage, which contributed to the low microbial counts observed throughout the storage period [78,79].

Furthermore, Table 8 displays the yeast and mold counts of the gummy samples stored under the same refrigerated conditions for 28 days. Similar to the TVC results, no yeast or mold growth was detected from day 0 to day 21, demonstrating robust stability. By day 28, yeast and mold counts were detectable, ranging from 0.66 to 4.67 CFU/g. The maximum counts were observed in RRP-100 (4.67 CFU/g) and GRP-100 (4.33 CFU/g), which were significantly higher (*p* < 0.05) than those of the other formulations. Conversely, the lowest counts were found in RRP-40 and GRP-60 (0.66 CFU/g).

The slight proliferation of microorganisms toward the end of the storage period may result from gradual oxygen exposure and continued metabolic activities within the food matrix [80]. However, the overall microbial, yeast, and mold counts remained heavily suppressed. In addition to refrigeration, this prolonged shelf life is likely fortified by the intrinsically acidic environment of the gummies. The formulations exhibited low pH values (approximately 3.26–3.77), driven by ingredients such as citric acid, which can disrupt cellular protein structures and create an unfavorable environment for the proliferation of most spoilage microorganisms, including yeasts and molds [81]. Overall, the results indicate that all gummy formulations maintained excellent microbiological quality until 21 days of refrigerated storage, while still remaining well within safe consumption limits by the end of the 28-day period.

## 4. Conclusions

The inclusion of KDML105 rice protein extracts in the formulations of gummy was effective in improving the nutritional, physicochemical and bioactive properties of the product. The substitution level of both fully ripe rice protein (RRP) and green rice protein (GRP) significantly increased the protein content of the gummy jellies and GRP-100 showed the highest level. The presence of these proteins changed the gel matrix and reduced hardness at high concentrations. However, a low substitution level (20%) maximized desirable textural properties such as springiness and chewiness, whereas moderate substitution (40%) optimally balanced cohesiveness and sensory acceptance, particularly in the RRP formulation. Furthermore, rice protein enrichment substantially improved the biological activities of gummy, particularly, GRP-100 was the best for the highest total phenolic content, total flavonoid content and antioxidant capacities than the control and RRP samples. Sensory evaluation revealed that protein fortified gummies were highly acceptable to consumers with GRP-100 receiving the highest overall preference score. Instrumental assessments via electronic nose and tongue confirmed that while higher levels of GRP slightly increased the bioactive-related bitterness and astringency, they simultaneously contributed to a highly complex aroma and improved richness. This robust aromatic profile actively functioned as a flavor modulator, creating an aroma-taste masking effect that seamlessly balanced the negative taste attributes. This sensory integration highlights the unique potential of green rice protein to enhance both functional and hedonic qualities without compromising consumer acceptance. The microbiological stability was good with low total microbial, yeast and mold counts during 28 days of refrigerated storage of the gummy jellies. Overall, GRP-100 demonstrated the most favorable sensory attributes and received the highest consumer acceptance among the formulations evaluated. In the mature rice protein group, the formulation containing 40% rice protein extract (RRP-40) showed the best sensory performance. These findings suggest that rice protein extracts, particularly those derived from green rice, have potential as plant-based ingredients for the development of gummy confections with acceptable sensory quality. Given the nutritional value of rice proteins and their generally recognized low allergenic potential, they may also offer opportunities for the development of functional confectionery products. However, further studies are needed to evaluate the bioavailability of key nutrients and bioactive compounds following consumption, as well as to verify the allergenic profile through appropriate in vitro, in vivo, and ultimately clinical investigations. In addition, future research should examine product stability during storage, optimize textural properties, and assess consumer acceptance in broader population groups.

## Figures and Tables

**Table 1:** Study of the formula of gummy supplemented with KDML 105 protein compared to the standard formula.

Samples	Water (%)	Rice Protein Extract (RRP, GRP) (%)	Other Ingredients (%)
Control	24.5	0	Sugar 33 Glucose syrup 33.5 Gelatin 8 Citric acid 1
20%	19.6	4.9	
40%	14.7	9.8	
60%	9.8	14.7	
80%	4.9	19.6	
100%	0	24.5	

Values are expressed as percentage of total formulation. The control sample represents the standard gummy formulation without rice protein extract substitution. RRP and GRP were used to replace water at levels of 20–100%. Other ingredients were kept constant in all formulations (sugar 33%, glucose syrup 33.5%, gelatin 8%, and citric acid 1%). RRP (Fully ripe KDML 105 rice protein extract), GRP (Green KDML 105 rice protein extract).

**Table 2:** Ten sensors and their corresponding sensitive substances.

Sensor Name	Aroma Type
W1C	Aromatic components
W5S	Nitrogen oxides
W3C	Aromatic and ammonia components
W6S	Hydrides
W5C	Aromatic components (short-chain alkanes)
W1S	Methyl compounds
W1W	Inorganic sulfides, terpenes
W2S	Alcohols, aldehydes, and ketones
W2W	Aromatic components (organic sulfides)
W3S	Long-chain alkanes

**Table 3:** Physical properties of gummies supplemented with rice protein extracts.

Samples	Color			TSS	pH	Syneresis
	L*	a*	b*			
Control	57.96 ± 0.63 ^cd^	−0.32 ± 0.01 ^b^	8.26 ± 0.68 ^ab^	31.93 ± 0.75 ^f^	3.49 ± 0.02 ^c^	2.92 ± 0.34 ^abc^
RRP-20	57.03 ± 0.85 ^cd^	−0.28 ± 0.03 ^b^	8.10 ± 0.30 ^abc^	34.83 ± 0.84 ^e^	3.27 ± 0.02 ^e^	1.78 ± 0.14 ^e^
RRP-40	56.06 ± 0.75 ^cd^	−0.29 ± 0.03 ^b^	7.86 ± 0.35 ^abc^	36.40 ± 0.90 ^d^	3.27 ± 0.03 ^e^	2.14 ± 0.30 ^de^
RRP-60	57.38 ± 1.68 ^cd^	−0.27 ± 0.16 ^b^	7.34 ± 0.52 ^cd^	36.62 ± 1.23 ^d^	3.26 ± 0.02 ^e^	2.8 ± 0.23 ^bc^
RRP-80	56.10 ± 0.90 ^cd^	−0.29 ± 0.07 ^b^	7.55 ± 0.23 ^bcd^	36.90 ± 0.89 ^cd^	3.53 ± 0.02 ^bc^	3.37 ± 0.19 ^a^
RRP-100	54.53 ± 0.75 ^d^	−0.22 ± 0.04 ^b^	7.08 ± 0.14 ^d^	38.37 ± 0.57 ^bc^	3.77 ± 0.03 ^a^	3.38 ± 0.19 ^a^
GRP-20	61.96 ± 1.45 ^bc^	0.12 ± 0.02 ^a^	8.30 ± 0.30 ^ab^	35.80 ± 0.79 ^de^	3.28 ± 0.01 ^e^	1.92 ± 0.02 ^e^
GRP-40	66.42 ± 1.99 ^ab^	0.13 ± 0.02 ^a^	8.42 ± 0.04 ^a^	37.00 ± 0.80 ^cd^	3.36 ± 0.02 ^d^	2.14 ± 0.49 ^de^
GRP-60	56.35 ± 1.92 ^cd^	0.09 ± 0.05 ^a^	7.64 ± 0.86 ^abcd^	38.26 ± 0.41 ^bc^	3.15 ± 0.01 ^f^	2.58 ± 0.39 ^cd^
GRP-80	69.50 ± 0.65 ^a^	0.18 ± 0.01 ^a^	8.00 ± 0.09 ^abc^	39.60 ± 0.80 ^b^	3.49 ± 0.02 ^c^	2.95 ± 0.18 ^abc^
GRP-100	69.80 ± 1.01 ^a^	0.13 ± 0.06 ^a^	7.94 ± 0.17 ^abc^	41.37 ± 0.60 ^a^	3.60 ± 0.12 ^b^	3.12 ± 0.22 ^ab^

All values represent the mean of three replicates ± SD. Statistical significance (*p* < 0.05) among means in a given column is indicated by distinct letters. RRP (Fully ripe KDML 105 rice protein extract), GRP (Green KDML 105 rice protein extract), TSS (Total soluble solids), numbers after sample names (20–100 respectively) are the amount of protein extract water replacement in different ratios to water ratio in the formula (%). L* = lightness (0 = black, 100 = white); a* = redness/greenness (+a* = red, −a* = green); b* = yellowness/blueness (+b* = yellow, −b* = blue).

**Table 4:** Proximate composition of gummy rice protein (g/100 g FW).

Samples	Carbohydrate	Proteins	Lipids	Ash	Moisture
Control	43.53 ± 0.41 ^b^	7.17 ± 0.25 ^j^	0.39 ± 0.01 ^g^	0.31 ± 0.02 ^h^	48.58 ± 0.39 ^a^
RRP-20	43.52 ± 0.11 ^b^	7.75 ± 0.06 ^i^	0.40 ± 0.01 ^g^	0.37 ± 0.02 ^fg^	47.94 ± 0.12 ^b^
RRP-40	44.00 ± 0.30 ^a^	8.43 ± 0.11 ^g^	0.44 ± 0.01 ^f^	0.42 ± 0.01 ^de^	46.69 ± 0.21 ^ef^
RRP-60	43.36 ± 0.11 ^b^	9.62 ± 0.02 ^e^	0.50 ± 0.01 ^e^	0.45 ± 0.01 ^c^	46.05 ± 0.13 ^g^
RRP-80	42.84 ± 0.06 ^c^	10.59 ± 0.01 ^d^	0.57 ± 0.02 ^d^	0.50 ± 0.01 ^b^	45.49 ± 0.05 ^h^
RRP-100	41.84 ± 0.10 ^d^	11.59 ± 0.08 ^b^	0.62 ± 0.01 ^b^	0.56 ± 0.01 ^a^	45.37 ± 0.03 ^h^
GRP-20	43.28 ± 0.06 ^b^	8.22 ± 0.02 ^h^	0.54 ± 0.01 ^d^	0.34 ± 0.02 ^gh^	47.6 ± 0.06 ^d^
GRP-40	42.63 ± 0.02 ^c^	9.26 ± 0.03 ^f^	0.59 ± 0.01 ^c^	0.39 ± 0.01 ^ef^	47.10 ± 0.01 ^c^
GRP-60	40.96 ± 0.03 ^e^	11.02 ± 0.02 ^c^	0.76 ± 0.01 ^a^	0.43 ± 0.01 ^cd^	46.81 ± 0.05 ^e^
GRP-80	40.68 ± 0.21 ^e^	11.52 ± 0.14 ^b^	0.75 ± 0.01 ^a^	0.51 ± 0.02 ^b^	46.52 ± 0.04 ^f^
GRP-100	40.19 ± 0.35 ^f^	12.63 ± 0.34 ^a^	0.76 ± 0.01 ^a^	0.57 ± 0.02 ^a^	45.84 ± 0.04 ^g^

All values represent the mean of three replicates ± SD. Statistical significance (*p* < 0.05) among means in a given column is indicated by distinct letters. RRP (Fully ripe KDML 105 rice protein extract), GRP (Green KDML 105 rice protein extract), numbers after sample names (20–100 respectively) are the amount of protein extract water replacement in different ratios to water ratio in the formula (%).

**Table 5:** Texture profile of gummies containing rice protein extract.

Samples	Hardness	Cohesiveness	Adhesiveness	Springiness	Gumminess (N)	Chewiness (N)
Control	39.42 ± 9.82 ^a^	0.88 ± 0.02 ^d^	−0.34 ± 0.29 ^b^	0.83 ± 0.03 ^e^	34.83 ± 8.52 ^b^	29.07 ± 6.61 ^c^
RRP-20	32.72 ± 3.39 ^bc^	0.88 ± 0.02 d^e^	−0.05 ± 0.02 ^a^	1.17 ± 0.07 ^a^	41.89 ± 2.31 ^a^	47.18 ± 1.55 ^a^
RRP-40	26.88 ± 8.76 ^cd^	0.92 ± 0.02 ^b^	−0.04 ± 0.01 ^a^	1.08 ± 0.05 ^c^	24.87 ± 7.77 ^c^	27.13 ± 8.56 ^c^
RRP-60	25.98 ± 9.04 ^d^	0.90 ± 0.01 ^bc^	−0.07 ± 0.02 ^a^	1.13 ± 0.02 ^b^	23.49 ± 7.7 ^cd^	26.74 ± 9.37 ^c^
RRP-80	25.65 ± 3.07 ^d^	0.85 ± 0.02 ^e^	−0.08 ± 0.05 ^a^	0.81 ± 0.04 ^e^	19.03 ± 1.67 ^de^	15.54 ± 2.04 ^d^
RRP-100	15.71 ± 0.58 ^e^	0.97 ± 0.01 ^a^	−0.06 ± 0.03 ^a^	1.03 ± 0.02 ^d^	15.24 ± 0.53 ^e^	15.83 ± 0.62 ^d^
GRP-20	35.42 ± 10.7 ^ab^	0.88 ± 0.02 ^d^	−0.06 ± 0.01 ^a^	1.19 ± 0.03 ^a^	31.31 ± 9.4 ^b^	37.49 ± 11.26 ^b^
GRP-40	27.32 ± 4.06 ^cd^	0.88 ± 0.02 ^cd^	−0.08 ± 0.03 ^a^	1.18 ± 0.04 ^a^	24.32 ± 3.78 ^cd^	28.84 ± 4.32 ^c^
GRP-60	24.18 ± 5.54 ^d^	0.87 ± 0.02 ^de^	−0.09 ± 0.03 ^a^	1.21 ± 0.04 ^a^	21.17 ± 4.48 ^cd^	17.78 ± 3.68 ^d^
GRP-80	24.03 ± 3.78 ^d^	0.86 ± 0.01 ^de^	−0.07 ± 0.03 ^a^	0.84 ± 0.02 ^e^	20.84 ± 2.83 ^cd^	25.43 ± 4.19 ^c^
GRP-100	22.82 ± 2.08 ^d^	0.87 ± 0.02 ^de^	−0.02 ± 0.00 ^a^	1.19 ± 0.05 ^a^	19.89 ± 1.33 ^cde^	23.81 ± 2.46 ^c^

All values represent the mean of three replicates ± SD. Statistical significance (*p* < 0.05) among means in a given column is indicated by distinct letters. RRP (Fully ripe KDML 105 rice protein extract), GRP (Green KDML 105 rice protein extract), numbers after sample names (20–100 respectively) are the amount of protein extract water replacement in different ratios to water ratio in the formula (%).

**Table 6:** Biological properties of gummies enriched with rice protein extract.

Samples	TPC (mg GAE/100 g DW)	TFC (mg QE/100 g DW)	DPPH (mg Vitamin C/100 g DW)	FRAP (mg Ferrous Sulfate/100 g DW)
Control	134.83 ± 13.50 ^h^	44.38 ± 1.58 ^j^	4.80 ± 2.34 ^i^	105.27 ± 2.54 ^h^
RRP-20	209.87 ± 10.68 ^g^	115.89 ± 1.78 ^i^	17.42 ± 1.41 ^h^	115.16 ± 0.62 ^g^
RRP-40	259.35 ± 14.31 ^f^	139.66 ± 2.96 ^h^	22.45 ± 0.30 ^g^	118.64 ± 2.96 ^g^
RRP-60	433.95 ± 11.48 ^e^	217.06 ± 2.96 ^f^	37.28 ± 0.10 ^d^	125.53 ± 1.57 ^f^
RRP-80	465.14 ± 9.05 ^d^	249.87 ± 2.63 ^e^	44.51 ± 0.19 ^b^	137.15 ± 1.31 ^e^
RRP-100	596.38 ± 3.75 ^c^	340.32 ± 14.40 ^c^	40.68 ± 0.54 ^c^	183.05 ± 1.57 ^b^
GRP-20	242.30 ± 6.66 ^f^	172.63 ± 3.25 ^g^	28.42 ± 0.41 ^f^	160.35 ± 2.82 ^d^
GRP-40	223.34 ± 3.62 ^g^	144.54 ± 5.48 ^h^	28.04 ± 0.52 ^f^	127.14 ± 2.13 ^f^
GRP-60	437.68 ± 17.85 ^e^	305.62 ± 20.72 ^d^	34.09 ± 0.81 ^e^	139.22 ± 2.60 ^e^
GRP-80	657.37 ± 7.83 ^b^	453.37 ± 1.47 ^b^	43.82 ± 0.34 ^b^	172.51 ± 2.59 ^c^
GRP-100	1099.45 ± 7.37 ^a^	486.08 ± 2.12 ^a^	47.70 ± 0.28 ^a^	192.24 ± 2.38 ^a^

All values represent the mean of three replicates ± SD. Statistical significance (*p* < 0.05) among means in a given column is indicated by distinct letters. GAE (gallic acid equivalents), QE (quercetin equivalents), Vitamin C and FeSO_4_ (Ferrous sulfate), RRP (Fully ripe KDML 105 rice protein extract), GRP (Green KDML 105 rice protein extract), numbers after sample names (20–100 respectively) are the amount of protein extract water replacement in different ratios to water ratio in the formula (%).

**Table 7:** Total plate count of gummy samples during refrigerated storage.

Samples	Total Plate Count (CFU/g of Sample)				
	0 Day	7 Days	14 Days	21 Days ns	28 Days
Control	nd	nd	nd	≤1.00	1.33 ± 0.57 ^ab^
RRP-20	nd	nd	nd	≤1.00	1.00 ± 1.00 ^b^
RRP-40	nd	nd	nd	≤1.00	1.66 ± 1.15 ^ab^
RRP-60	nd	nd	nd	≤1.00	1.67 ± 0.57 ^ab^
RRP-80	nd	nd	nd	≤1.00	1.33 ± 0.57 ^ab^
RRP-100	nd	nd	nd	≤1.00	2.00 ± 1.00 ^ab^
GRP-20	nd	nd	nd	≤1.00	1.00 ± 0.01 ^b^
GRP-40	nd	nd	nd	≤1.00	1.33 ± 0.57 ^ab^
GRP-60	nd	nd	nd	≤1.00	1.33 ± 0.57 ^ab^
GRP-80	nd	nd	nd	≤1.00	1.33 ± 0.57 ^ab^
GRP-100	nd	nd	nd	≤1.00	2.66 ± 0.57 ^a^

All values represent the mean of three replicates ± SD. Statistical significance (*p* < 0.05) among means in a given column is indicated by distinct letters, nd = not detectable, ns = non-significant. RRP (Fully ripe KDML 105 rice protein extract), GRP (Green KDML 105 rice protein extract), numbers after sample names (20–100 respectively) are the amount of protein extract water replacement in different ratios to water ratio in the formula (%).

**Table 8:** Yeast and mold count of gummy samples during refrigerated storage.

Samples	Total Yeast and Mold (CFU/g of Sample)				
	0 Day	7 Days	14 Days	21 Days	28 Days
Control	nd	nd	nd	nd	1.33 ± 0.57 ^b^
RRP-20	nd	nd	nd	nd	0.67 ± 0.57 ^b^
RRP-40	nd	nd	nd	nd	0.66 ± 0.57 ^b^
RRP-60	nd	nd	nd	nd	1.00 ± 1.01 ^b^
RRP-80	nd	nd	nd	nd	1.66 ± 1.52 ^b^
RRP-100	nd	nd	nd	nd	4.67 ± 1.52 ^a^
GRP-20	nd	nd	nd	nd	1.33 ± 0.57 ^b^
GRP-40	nd	nd	nd	nd	1.67 ± 0.57 ^b^
GRP-60	nd	nd	nd	nd	0.67 ± 0.57 ^b^
GRP-80	nd	nd	nd	nd	1.67 ± 0.57 ^b^
GRP-100	nd	nd	nd	nd	4.33 ± 1.15 ^a^

All values represent the mean of three replicates ± SD. Statistical significance (*p* < 0.05) among means in a given column is indicated by distinct letters, nd = not detectable. RRP (Fully ripe KDML 105 rice protein extract), GRP (Green KDML 105 rice protein extract), numbers after sample names (20–100 respectively) are the amount of protein extract water replacement in different ratios to water ratio in the formula (%).

## Data Availability

The original contributions presented in this study are included in this article. Further inquiries can be directed to the corresponding author.
